# Temporal understanding of human mobility: A multi-time scale analysis

**DOI:** 10.1371/journal.pone.0207697

**Published:** 2018-11-27

**Authors:** Tongtong Liu, Zheng Yang, Yi Zhao, Chenshu Wu, Zimu Zhou, Yunhao Liu

**Affiliations:** 1 Tsinghua University, Beijing, China; 2 University of Maryland, College Park, United States of America; 3 Swiss Federal Institute of Technology in Zurich, Zurich, Switzerland; CSIRO, AUSTRALIA

## Abstract

The recent availability of digital traces generated by cellphone calls has significantly increased the scientific understanding of human mobility. Until now, however, based on low time resolution measurements, previous works have ignored to study human mobility under various time scales due to sparse and irregular calls, particularly in the era of mobile Internet. In this paper, we introduced Mobile Flow Records, flow-level data access records of online activity of smartphone users, to explore human mobility. Mobile Flow Records collect high-resolution information of large populations. By exploiting this kind of data, we show the models and statistics of human mobility at a large-scale (3,542,235 individuals) and finer-granularity (7.5min). Next, we investigated statistical variations and biases of mobility models caused by different time scales (from 7.5min to 32h), and found that the time scale does influence the mobility model, which indicates a deep coupling of human mobility and time. We further show that mobility behaviors like transportation modes contribute to the diversity of human mobility, by exploring several novel and refined features (e.g., motion speed, duration, and trajectory distance). Particularly, we point out that 2-hour sampling adopted in previous works is insufficient to study detailed motion behaviors. Our work not only offers a macroscopic and microscopic view of spatial-temporal human mobility, but also applies previously unavailable features, both of which are beneficial to the studies on phenomena driven by human mobility.

## Introduction

People are curious about their movement patterns and have been diligently exploring the basic laws behind their mobility for a long history. The study of human mobility plays an important role in many subjects of science[1], such as physics, biology, anthropology, demography, sociology, history, etc. Human mobility is composed of a large population of free-will and autonomous decision-making individuals; and it is also influenced by many unknown factors and their interaction[1]. As a result, the characterization of human mobility is extremely difficult, attracting many researchers engaged in the study of this area in the past decades.

The effective measures of mobility of a large-scale population are absent for a long time. In the early stage of study, based on the measurements of albatrosses, monkeys and marine predators, some researchers study the general pattern of animal mobility[2–4], which can be used as an approximation of human mobility. A subsequent trend of research works utilizes indirect measurements of human movement as their primary data. Typical examples include tracking bank note circulation[5], monitoring taxi trajectory[6, 7], documenting public transit data[7], and collecting user geo-tagged logins of online social networks[8]. Particularly, the study on bank note circulation suggests that human trajectories are well modelled as a random walk with fat-tailed displacement and waiting-time distribution, given that bank note dispersal is a proxy for human movement[5]. All these tactful means, however, introduce self-insurmountable shortcomings of indirect measures, e.g., sampling bias, proxy complication, and the interaction of unknown factors.

In recent years, the availability of telecommunication big data capturing aspects of human mobility has given a new empirically driven momentum to the subject. Cellphone data (specifically, Calling Description Records, CDRs) is collected by mobile operators for billing purpose originally. CDR includes calling records, as well as the time, location, duration, and other information associated with each phone call. The use of CDR enables tracking individual movements of a large population of mobile phone users, which made a milestone progress of human mobility research. Making full utilization of these data has demonstrated its great potentials to a broad range of novel applications, including crowd flow forecasting[9–11], taxi demand prediction[12], public transportation planning[7, 13, 14], urban planning[15, 16], etc., which are the basis to provide effective, real-time, and intelligent city management and services. Existing studies show that human mobility can be described by a number of quantitative characteristics, e.g. jump size and gyration radius, and it can be best modelled by Lévy flight[17, 18] or Continuous-Time Random-Walk (CTRW)[19, 20] models, two classical and well-studied modeling frameworks in the random-walk community[21]. However, those findings are observed when sampling rate is low, which results in incomplete human movement. Previous works intentionally select a specific group of users whose call frequency is greater than 0.5 times per hour (a phone call in every 2 hours on average) within a certain time period, which reluctantly filters out a vast majority of available participants being investigated[1, 22]. Even for those preserved users, 2-hour time span of consecutive location samples is too coarse-grained and may omit individual movement lasting less than two hours, leading to underestimated range of movement, inaccurate waiting-time estimation, and low rate of convergence of statistics and model parameters. Recently, the extensive use of GPS enables researchers to study human mobility at a finer granularity than before and some works try to reveal how temporal resolution impacts the observations of human mobility by GPS[23, 24]. However, due to privacy issues, GPS data sets usually contain a limited number of people and conclusions based on GPS may be biased. Human mobility patterns of a large group of people at high time resolution are still absent so far. Meanwhile we have no idea how time resolution influences the statistical model of mobility of the crowd.

In the mobile Internet era, people’s online activities, like sending an instant message, browsing websites, watching online videos, playing mobile games, even regular background application data exchange and automatic update, has partly taken place of traditional phone calls and become increasingly prevailing; thus providing much richer and denser Internet access logs than CDR[25–27]. Mobile Flow Records (MFRs), system logs collected by mobile operators, document such online activities of data cellular networks, including flow-level wireless-specific resource-usage information and the relation of traffic to individual subscriptions and devices. Regarding to human mobility, MFR provides much higher time-resolved user locations and captures more detailed motion behavior than CDR.

In this study, we analyzed and modeled human mobility based on two data sets of MFR, capturing about 3.5 × 10^6^ users for 1 week and 1.4 × 10^6^ users for 5 weeks respectively. Taking advantage of high time-resolved records provided by MFR, we resampled from original data sets to get data sets under different time scales (sampling interval ranging from 7.5min to 32hour). The results of our experiments indicate that the time scale does influence the fitting parameters of mobility model, which indicates a deep coupling of human mobility and time. Specifically, our findings suggest a decreasing trend of diffusion rates along with increasing sampling intervals. Our results also clarify that time resolution may be an explanatory variable for model inconsistency and variability in previous works[1, 21, 22, 24, 28, 29].

MFR provides fine-grained location information for large-scale populations, which enables researchers to dig deeper into the human mobility. We believe that it will be an important proxy to study human behaviors in the future. Despite the advantages, MFR also has some limitations. For example, the spatial resolution of MFR is determined by the density of cell towers and is still less accurate than that of GPS. The sampling of MFR is passive and nonuniform[25].

## Results

### Data sets and measures

**Fig 1:**
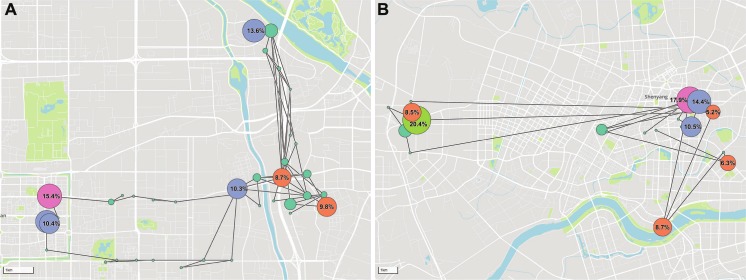
Mobility networks of two typical users. The circles in the map denote the recorded locations of users based on MFR. The size of each circle, as well as the associated percentage, expresses the weight of each location, which is determined by the waiting time at every location. **A**, User 1’s trajectory presents regular weekday commute between his workplace and his home, and a weekend route to scenic spots. **B,** User 2’s trajectory consists of 3 frequently visited locations: home, workplace, and entertainment, each of which is with different weights. The labels of locations (e.g., home, workplace, entertainment, scenic spots) are speculated from city Point of Interests (POIs) information, time and duration of stays, frequency of visits, and dates (holiday and what day of a week). Visualization of users’ trajectories can be queried from an interactive website http://tns.thss.tsinghua.edu.cn/humanmobility.

We used two data sets of MFR to explore the mobility pattern of individuals. The first (D1) consists of the mobility patterns recorded over one-week period for 3,542,235 anonymized mobile phone users in Xi’an, a metropolitan locating in the west part of China. To make sure that the obtained results were not affected by particular city-specific characteristics, we also studied a data set (D2) that captured the locations of 1,387,448 mobile phone users over 5 weeks, in Shenyang, a major city in northeastern China. In both data sets, the spatial resolution was determined by the local density of more than 6,062 cell towers for D1 and 1,548 cell towers for D2, registering movement only when a user moved between areas serviced by different towers. The average service area of each tower was about 1.68 km^2^ and 1.03 km^2^ for D1 and D2, respectively. Fig 1 shows the mobility networks of two typical users deduced from MFR data. See detailed information about MFR and datasets in S1 Table.

**Fig 2:**
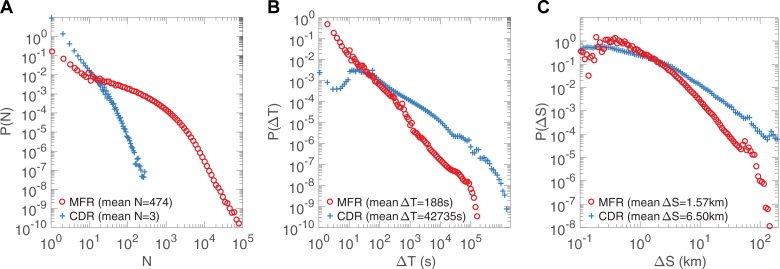
Comparison of MFR (D1) and CDR. **A**, The distribution of daily number of records *N* per person. **B,** The distribution *P(ΔT)* of interevent time *ΔT*. **C,** The distribution *P(ΔS)* of interevent distance *ΔS*. In B**, C**, red lines (MFR) are significantly steeper than blue lines (CDR), indicating that MFR provides finer spatial and temporal granularity than CDR.

In addition, we used a CDR data set to compare the efficiency of MFR and CDR. The CDR data set collects records of 572,707 users in Urumqi, another Chinese city for 1 month. Compared with the CDR data set used in previous work[1], they share similar interevent time distributions. This indicates that CDR data sets collected from different cities are likely to have similar patterns. Therefore, although the CDR data set was not collected from the same city as MFR, they are still comparable. Fig 2A shows the distribution of daily number of records *N* per person and we found that MFR provided much more mobility information that CDR for most people. To make a comparison of their spatial-temporal granularity, we studied the distribution of interevent time *ΔT* and interevent distance *ΔS*, the time interval and distance between consecutive communication records of the same user. For the MFR data set, the sample size of *ΔT* is 243,439,240 and that of *ΔS* is 25,541,472; for the CDR data set, the sample size *ΔT* is 4,810,118 and that of *ΔS* is 2,574,428. As is shown in Fig 2B and 2C, *ΔT* and *ΔS* of MFR and CDR followed a ‘bursty’ pattern but *P(ΔT)* and *P(ΔS)* of MFR are significantly steeper than those of CDR. The average time interval is 188 seconds for MFR and about 12 hours for CDR. Previous works only kept users whose call frequency was larger than 0.5h^-1^ to ensure trajectory completeness[1, 22]. However, this process filters a large number of users and the 2-hour call interval is still large.

Since the numbers of users in these two data sets are different (3.5 million for MFR and 0.5 million for CDR), these may affect the results in Fig 2. To exclude the influence of data set size, we down-sampled the two data sets of MFR and CDR to make them contain the same number of individuals. Table 1 shows the results. The averages of these metrics are stable under different sample sizes and are consistent with the corresponding averages in Fig 2. Therefore, the current results are enough to show the differences between MFR and CDR. We can conclude that MFR is a better proxy with finer spatial and temporal granularity than CDR for human movement and it enables us to explore human mobility under multi-time scales.

**Table 1:** Comparison of MRF(D1) and CDR at different sample sizes.

Sample Size		50,000	100,000	500,000
**N**	**CDR**	**3.39**	**3.37**	**3.42**
	**MFR**	**468.23**	**468.96**	**473.81**
**ΔT (s)**	**CDR**	**43166.71**	**43535.76**	**42765.44**
	**MFR**	**187.35**	**187.65**	**187.27**
**ΔS (km)**	**CDR**	**6.76**	**6.57**	**6.50**
	**MFR**	**1.57**	**1.57**	**1.57**

The averages of daily number of records *N*, interevent time *ΔT*, and interevent distance *ΔS* at different sample sizes. These averages are stable under different sample sizes and are consistent with the corresponding averages in Fig 2.

To understand mobility behaviors, we investigated several features (e.g., jump size, radius of gyration, waiting time, speed, and transportation mode) and analyze their variation trend under various time scales (sampling intervals ranging from 7.5-min to 32-hour). In the following sections, we use “high sampling rates” and “small sampling intervals” to mean that the locations of the individual are recorded frequently (usually sampling intervals are less than 30 minutes), while “low sampling rates” and “large sampling intervals” mean that the sampling process is relatively less frequent (usually sampling intervals are larger than 2 hours).

### Jump size under multi-time scales

**Fig 3:**
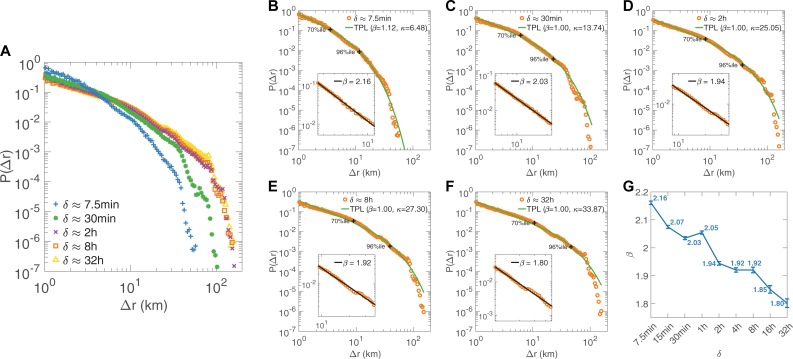
Displacement of human mobility (D1, see S2 Fig for D2). **A**, The distribution of displacement *P(Δr)* under time scales *δ* ≈ 7.5min, 30min, 2hour, 8hour, 32hour. **B-F**, The solid lines (green and blue) indicate a truncated power law and a log-normal distribution with best fitting parameters, respectively. The insets show the best power law fitting for the tails (from 70%ile to 96%ile). G, The variation trend of fitting parameter *β* with time scale *δ* with standard deviation as error bar.

We measured the jump size, the distance between user’s positions at consecutive data access records, capturing more than 10^6^ displacements for the D1 and D2 under each time scale (except for 32hour-sampling having more than 10^5^ samples). We re-sampled the raw MFRs with the sampling intervals of *δ* ≈ 7.5min, 15min, 30min, 1hour, 2hour, 4hour, 8hour, 16hour, 32hour. And the corresponding numbers of samples for every sampling interval are listed in Table 2. We found that the distribution of jump size (*Δr*) over all users is well approximated by a truncated power-law with exponential cut-off (denoted as TPL(*β*, *κ*) in Fig 3):

$$P(\Delta r)\propto {\Delta r}^{-\beta }\;\exp\;\left(-\frac{\Delta r}{\kappa }\right)$$ (1)

with the fitting parameters, however, behaving differently at different time resolutions. Since the results are consistent under all time scales, Fig 3 only shows the cases of *δ* ≈ 7.5min, 30min, 2hour, 8hour, 32hour (see other cases in S1 Fig). We further zoomed in for a closer look at the tail distribution of measurements from 70%ile to 96%ile and found that they are best modeled by power law distribution, shown in the insets of Fig 3B–3F, for time scales of *δ* ≈ 7.5min, 30min, 2hour, 8hour, 32hour, respectively. This conclusion also holds for any subset of tails, e.g., from 80% to 95%. It is worth mentioning that, for sufficiently large time scales, the frequency distribution of *Δr* collapses when *Δr* ≥ 80km, which seems to be related to the scale of urban areas. Note that the range of our data set covers both urban and rural areas of a city.

**Table 2:** Number of samples for each sampling interval.

Sampling interval *δ*	Number of samples
7.5 minutes	7,567,233
15 minutes	8,258,692
30 minutes	7,006,878
1 hour	5,071,176
2 hours	3,498,286
4 hours	2,226,450
8 hours	1,347,365
16 hours	702,218
32 hours	508,482

The corresponding numbers of samples for every sampling interval.

Our findings about *Δr* include two aspects. First, the mobility patterns, deriving from the same datasets but with different time scales, are best described by various values of *β*, indicating that time scales (sampling rates) do influence mobility model. Second, the values of *β* range from 2.16 to 1.80 when sampling intervals rise from 7.5 minutes to 32 hours. As *β* is a metric of diffusion speed, human movement presents a certain extent of purposefulness when *δ* < 2 hours, in accord with our intuition of a commuting trajectory with a destination. In contract, when *δ* ≥ 2 hours, the extent of purposefulness weakens. The overall trend of diffusion speeds is decreasing along with time scales.

### Radius of gyration

**Fig 4:**
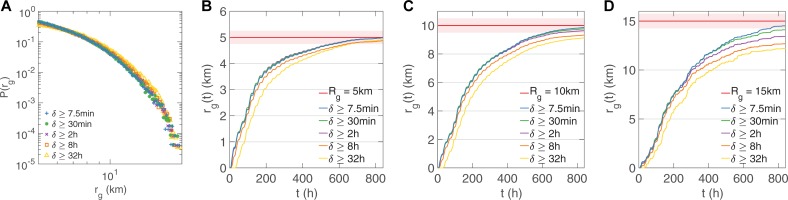
Gyration radius of human mobility (D2, see S3 Fig for D1). **A**, The distribution of gyration radius *r*_*g*_ under time scales *δ* ≥ 7.5min, 30min, 2hour, 8hour, 32hour. We further divided all users into 3 groups according to their final gyration radius *r*_*g*_*(T)* during the whole observation period *T*. **B-D,** show the convergence speeds of *r*_*g*_ of different user group *r*_*g*_*(T)* = *R*_*g*_ ± 0.05*R*_*g*_ and *R*_*g*_ = 5km, 10km, 15km, respectively.

When studying human mobility, the radius of gyration (*r*_*g*_) is another important statistic that indicates the characteristic distance travelled by a person during a period. We show the distributions of *r*_*g*_ under time scales ranging from 7.5min to 32hour (*n* = 369,539 samples) and depict the results only for *δ* ≥ 7.5min, 30min, 2hour, 8hour, 32hour in Fig 4A due to visual clarity of figure. The results for intermediate time scales are consistent. To our surprise, although time scales vary greatly, all of them are able to capture the tail distribution of *r*_*g*_ characteristically. One reason for this may be the sufficiently long observation period, 35 days for D2. Regarding to user groups of different mobility modes, however, we hypothesized that sampling rates have a notable impact on moving trajectory description, which is reflected in the measurement of *r*_*g*_. And we tested it by measuring the time dependence of gyration radius for users whose gyration radius would be considered small (*r*_*g*_*(T)* ≈ 5km, *n* = 21,264 samples), medium (*r*_*g*_*(T)* ≈ 10km, *n* = 3,793 samples), and large (*r*_*g*_*(T)* ≈ 15km, *n* = 255 samples) at the end of our observation period. As shown in Fig 4B, 4C and 4D, we observed that 1), for the group of almost-static users, only sampling rates higher than 8-hour (considerably low sampling rates) are able to accurately estimate *r*_*g*_; 2), for the group of moderately mobile users, different sampling rates begin to behave differently, and the lower the sampling rates, the more underestimated *r*_*g*_; 3), for highly mobile users, the above-mentioned phenomenon emerges earlier at around 200 hours and finally lower sampling rates result in significant deviation from ground truth. The results from D1 (see S3 Fig) also verify our statement.

Another thing worth to mention is that, previous work[22, 28] concludes that the time dependence of the average radius of gyration is better approximated by a logarithmic increase in a large time scale (months). Our results show the detailed description of how *r*_*g*_ increases in the first few days, which is unlikely a logarithmic increase but a linear increase with different fitting parameters. The linear increase lasts for about a week, indicating that for the majority, the mobility patterns change a lot in the first week and gradually tend to stabilize. This means that people’s behaviors are likely to be different on different days of a week and be similar at the same time of different weeks. This conclusion is consistent with the fact that our life is basically in a one-week cycle. Due to the short duration of D1 (see S3 Fig), the process of how *r*_*g*_ converges is not complete. Thus, whether and how a city infects *r*_*g*_ of its citizens needs further research.

### Time related statistics

**Fig 5:**
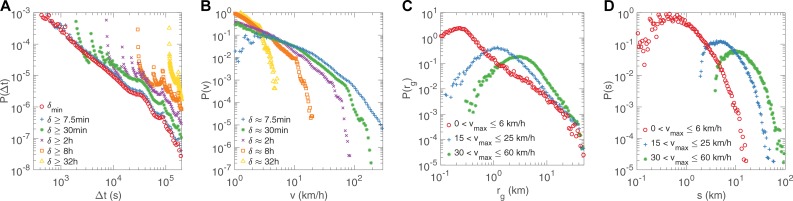
Time related statistics of human mobility (D1, see S4 Fig for D2). **A**, The distribution of waiting time *Δt* under time scales *δ*_*min*_ and *δ* ≥ 7.5min, 30min, 2hour, 8hour, 32hour. **B**, The distribution of moving speed under time scales *δ* ≈ 7.5min, 30min, 2hour, 8hour, 32hour. **C,** The distribution of radius of gyration for 3 user groups with different max speeds 0 < *v*_*max*_ ≤ 6km/h, 15 < *v*_*max*_ ≤ 25km/h, 30 < *v*_*max*_ ≤ 60km/h during the whole observation period *T*. **D,** The distribution of moving distance for each group during morning rush hour (7am to 10am).

We measured *P(Δt)*, where the waiting time *Δt* is defined as the time a user spent at one location. It is apparent that the higher time resolution, the more accurate waiting time measurements. But we have no idea what sampling rate is sufficient to capture waiting time and how sampling rates influence the measurements. One specific case is that, due to low sampling rate, it is extremely difficult to document user’s waiting time using CDR data. We first show the distribution of *Δt* under various sampling rates *δ*_*min*_ (*n* = 11,323,385) and *δ* ≥ 7.5min (*n* = 4,911,081), 30min (*n* = 2,695,818), 2hour (*n* = 1,501,153), 8hour (*n* = 849,327), and 32hour (*n* = 404,393) in Fig 5A. Note that *δ*_*min*_ is the highest sampling rate we can achieve via using all time-stamped data (See the method part for details). There seems a threshold of sampling interval of *δ* ≥ 7.5min, under which the waiting time can be accurately captured and obeys a power law distribution. In contrast, the waiting time measurements at larger sampling intervals are meaningless due to unreasonable repeating patterns of distribution caused by coarse sampling, shown in Fig 5A. Different from the previous result[21], the distribution of *Δt* follows a power-law model of *P(Δt)∼|Δt|*^*-β*^ with exponent *β* = 1.5708 ± 0.0004 (mean ± standard deviation), rather than a power-law model with *β* = 1.8 ± 0.1 based on CDR or geo-tagged signed up for a location-based service[21]. This inconsistency can be attributed to sampling bias: the time unit used in previous work was one hour, which was too large to capture an accurate duration of a stay at a specific location; thus, *P(Δt)* tends to be underestimated for large *Δt* and overestimated for small *Δt*, leading to a larger exponent.

Owing to low-density sampling, most previous research works based on CDR did not study a critical metric of motion, the moving speed, which is important not only for understanding how human move, but also for a series of real-world applications, e.g., crowd flow prediction, traffic control and engineering, public transportation planning, road planning, urban planning, etc. Based on MFR, we were able to characterize moving speed distribution for a large population in an unprecedented granularity. The moving speed *v* is defined as *v = Δr/τ*, where *Δr* and *τ* are the displacement and time of each motion, respectively.

We show the distribution of moving speed under time scales *δ* ≈ 7.5min (*n* = 7,567,233), 30min (*n* = 7,006,878), 2hour (*n* = 3,498,286), 8hour (*n* = 1,347,365), and 32hour (*n* = 508,482) in Fig 5B. We found that, as the highest sampling rate, 7.5-min sampling best reflects the speed distribution we see in our real life; besides, the 30-min sampling yields approximate estimation, except for overestimated frequency of low speed cases; 1-hour sampling performs marginally acceptably (not shown due to space limit in the figure). However, low sampling rates (2-, 8-, 32-hour) fail to capture the moving speeds larger than around 10km/h (not a high speed at all). Particularly, taking the 7.5-min sampling (blue line) as an example, it captures human moving speeds ranging from 0-100km/h, which further can be used to indicate the forms of urban transportation (e.g., walking, cycling, riding or driving), a unique characteristic of human motion, rather than animals. From another aspect, for a high driving speed >60km/h, 7.5-min sampling shows around 5.25% of people traveling are probably by car or public transportation; in contrast, this portion slumps to a completely ignorable level of 10^−6^ (roughly several individuals) if sampling at every 2 hours, which is unreasonable.

From our point of view, the reasons for phenomena are twofold. First, the displacement length is likely to be shorter than the actual trajectory length and the larger the sampling interval is, the more significant the difference is. Second, when sampling intervals are getting larger, there may be more travels and stops between two adjacent samples and this can lead to a lower average speed. Therefore, 2-hour sampling adopted in previous works is insufficient to study some critical metrics of motion, including waiting time, traveling speed, and other time- or speed-related quantitative features.

To further uncover the relationship between the movement statistics and speed of motion, we grouped people by their traveling speeds and investigated the characteristics of gyration radius *r*_*g*_ and route distance Δ*s* for every group. The criteria of user classification according to speed are as follows: 1, users whose mainly transportation mode is walking with a maximum speed 0 < *v*_*max*_ ≤ 6km/h (*n* = 13,807); 2, users who move moderately fast with a maximum speed 15 < *v*_*max*_ ≤ 25km/h (*n* = 22,516), indicating a mixed transportation of walking, bicycling, riding, or driving; 3, users who move adequately fast with a maximum speed 30 < *v*_*max*_ ≤ 60km/h (*n* = 38,473), indicating a transportation mode of riding or driving vehicles.

From Fig 5C, the average *r*_*g*_ of such 3 groups of people are 1.41km, 3.15km and 5.44km, respectively. The values of *r*_*g*_ seem to be underestimated considering the speed. The reason is that when computing *r*_*g*_, the waiting time at location *i* is used at the weight of the squaring of the distance between location *i* and the center of mass. For most people, a few specific places (e.g., home or working site) take up a large portion of their time and accordingly obtain more weights in the computation of *r*_*g*_, while some faraway places have lower weight.

To overcome such shortcomings, we show the relationship between the route length and speed, where the route length is the total distance of consecutive sightings along a route. As shown in Fig 5D, the average route lengths during morning rush hour (7am to 10am) of 3 groups are 1.38km, 8.30km and 16.82km, suggesting that the commuting distances vary with different transportation modes. Previous studies on large population mobility are either unable to distinguish transportation modes or based on a single data source of a specific transportation mode. By exploring several novel features (e.g., motion speed, duration, and trajectory distance) based on MFRs, we show that the mixture of transportation modes is another important dimension contributing for the diversity of mobility behaviors.

## Discussion

We have shown that mobility models, including model parameters and goodness of fit, are significantly influenced by sampling rates. It suggests that previous works that modeled their measurements by TPL with different values of *β* may have no direct conflict of each other due to different sampling rates. The fundamental reason for the differences is that observations with one sampling rate are insufficient to fully describe human mobility. Besides, we have found that the diffusion speeds show a downward trend when sampling intervals increase. Although the values of *β* ranging from 2.16 to 1.80 comply with Lévy Flight model, the decreasing trend of *β* implies purposeful human movement, contradicting with the random direction selection assumption of Lévy Flight model[30]. All observations above indicate that one constant model cannot explain human movement properly and a more appropriate one should vary under different time scales. It is worth mentioning that, for the crowds of a city, the mobility model is also impacted by the scale of urban areas of the city.

In addition to time scales, we have pointed out that movement statistics are highly relative to moving speeds too. When people move at different speeds, the patterns of their mobility present completely different characteristics. For example, we have found that in terms of commuting, higher speed usually means longer commuting distance and therefore people with higher speeds usually have larger gyration radius. Since different speeds usually mean different transportation modes, how the heterogeneity of transportation modes affect the observed mobility patterns deserves extensive research.

By studying MFR, we explored human mobility patterns at a finer granularity. Moreover, MFR enables us to find out which time scale is suitable for a particular measurement job. When measuring the distribution of *r*_*g*_, sampling intervals are concerned with space range and time duration. If the crowds are in a large area and the measurement duration is limited, a high sampling rate is necessary to ensure the effectiveness. When sampling rate cannot be increased for either technical or non-technical reasons, the measurement should last long enough to guarantee the convergence of *r*_*g*_. In order to explore the patterns of moving speed, sampling intervals have to be less than 30 minutes. The typical sampling rate of CDR, 0.5h^-1^, is widely adopted[1, 22] but insufficiently good to estimate the proportion of high speed accurately. Therefore, data sources with finer granularity, like MFR and GPS, are required.

## Methods

### Data description

Every time when one mobile phone accesses the Internet, the records of cell tower ID, timestamp and other necessary information about the data traffic flow are collected by mobile operators for billing and operational purposes. We call such records as mobile flow records. In the mobile Internet era, online activities have replaced traditional phone calls and text messages to become the main usage of mobile phones. Therefore, MFRs offer a better proxy to study human mobility than CDRs.

In our experiment, we used two anonymized MFR data sets, collected by a major cellular carrier in two big cites of China, to analyze and model human mobility. The first set (D1) captured 3,542,235 anonymized cellphone users in Xi’an, a central Chinese city with an area of 10,108 km^2^ over one-week period. The second set (D2) contained the mobility pattern of 1,387,448 anonymized individuals in a 40 × 40 km urban area of Shenyang, a major city in northeast China, recorded for 5 weeks. A typical MFR consists of a unique anonymized user ID, a corresponding cell tower ID, a timestamp of the creation time, an APP ID, a device type ID and other information about the uplink and downlink traffic (see S1 Table for detailed column description). For a device, the location of its connected cell tower is an effective approximation of its location and we reconstructed its trajectory based on the time-ordered list of cell towers. Since the location of cell tower was considered as an approximated location of each record, the spatial resolution was determined by the local density of cell towers. The quantity of cell towers for the D1 set is 6,062 and for the D2 set is 1,548. The average service area of each tower is about 1.68 km^2^ and 1.03 km^2^ for D1 and D2 respectively.

### Re-sampling

To explore the mobility pattern of individuals under different time scales, re-sampling is necessary to convert an original data set to another one under a lower sampling rate. In our experiment, we implemented two kinds of re-sampling methods. At the beginning of both methods, the time-ordered list of mobile records was sorted for each individual. In the first method, starting from the first record, we kept one record if the time interval between it and the last kept record is greater than or equal to the given interval *δ*_*t*_ and dropped one otherwise. By this method, we could get a complete trajectory but under a lower sampling rate for each individual. The result of this method is denoted as *δ* ≥ *δ*_*t*_ in the corresponding figure legend. This method was used in the experiment when calculating gyration radius and waiting time distribution of each trajectory. The second method only kept records if they were separated by an interval *δ*_*t*_ ± 0.05 *δ*_*t*_. Different from the first one, the second method could not retain the integrity of each trajectory but it had the advantage that intervals between consecutive records were similar (≈ *δ*_*t*_). The result of the second method is denoted as *δ* ≈ *δ*_*t*_ in the corresponding figure legend. We used this method when calculating jump size, moving speed and route length of each individual. In addition, the denotation *δ*_*min*_ in legends means that the corresponding characteristic was computed directly upon the original data set without any re-sampling.

### Distribution fitting

In our study, we applied the method of maximum likelihood to estimate parameters for the fit to empirical data[31, 32]. Accurate parameter estimates can be derived by maximizing the likelihood functions. Particularly, we used the *powerlaw* Python package to conduct the fitting[33]. The *powerlaw* package is a statistical software to analyze a variety of probability distributions, including basic power-law, truncated power-law with exponential cut-off and log-normal. It provides functions to fit observed data to a specific distribution. However, this tool does not provide the information of standard deviations and confidence intervals. We derived the 95% confidence intervals by the Fisher Information and implemented our distribution fitting procedure based on the functions of the *powerlaw* package by Python.

### Statistical tests

Given the power-law distribution observed in the data sets, it is important to test if the best fit is statistically consistent with the corresponding data. The data sets in our study consist of millions of displacements and therefore traditional statistical tools, which are designed to deal with limit data, are less important[1]. We took the statistical tools, Kolmogorov-Smirnov (KS) test, to examine the goodness of the fit. By performing the KS test, we could determine whether the empirical data comes from the best fits.

The KS statistics is an indicator that shows to what degree two distributions are the same. Two kinds of KS statistics were used in our experiment to conduct the KS test. The first is the standard KS statistics, denoted as *KS*, which is defined as:

KS=max(|F−P|) (2)

where *F* and *P* are the cumulative distributions of the fit and data respectively. Since the standard KS statistics is not sensitive on the edges of the cumulative distribution, we introduced the weighted KS statistics *KS*_*W*_, which is defined as:

$$KS_{W}=max\frac{\left|F-P\right|}{\sqrt{P(1-P)}}$$ (3)

In the test, the null hypothesis is that the empirically observed distributions come from its best fitted distribution. Our general approach was to generate synthetic data starting from the fitted distribution and then perform KS test to see if the empirical data behave as well as the synthetic data. For this, we computed the *KS* and *KS*_*W*_ statistics between the empirical data and its fit, the synthetic data generated and the fit respectively. If the empirical data behave as good as or better than the synthetic data, that is, the *KS* and *KS*_*W*_ for the empirical data are not greater than those for the synthetic data, it means that the empirical data can be the result of its fit. For each pair of data and fit, we generated 1,000 synthetic data sets to test the goodness of this fit and used *p*-value to summary the results of the KS test. Here the p-value is defined as the probability that the *KS* and *KS*_*W*_ statistics of the synthetic data were smaller than those of the empirical data and it represents the probability that the empirical data was the result of the fit. A *p*-value close to 1 indicates the consistency between the empirical data and its fit and one close to 0, specifically smaller than 0.01, means the empirical data cannot come from this fit.

S5 Fig compares the *KS* statistics of the empirical tail distribution of jump size (like the insets of Fig 3B–3F) with those for 1,000 distributions of synthetic data generated from corresponding fitting distribution. The *p*-values for the nine fits are 1.00, 1.00, 1.00, 1.00, 1.00, 0.46, 0.04, 0.00 and 0.03 respectively. The fits under all time scales passed the *KS* test except for 16-hour. From the inset of S1D Fig, it is obvious that this was caused by the roughness at *Δr ≈ 25km*. The same phenomena can be found when other sample time *δ ≥ 2h* but they are more slight. We believe that the root cause lies in the difference of human mobility between the urban core area with others. S6 Fig shows the same for the *KS*_*W*_ test and the *p*-values are all 1.00 under 9 time scales. In this case, all the fits passed the test, including the fit of 16-hour. Therefore, we can conclude that the power law offers a good approximation of the observed tail distribution of jump size and the scaling parameters *β* are meaningful.

### Computation of gyration radius

We used the radius of gyration *r*_*g*_ defined as[1, 28, 34]:

$$r_{g}=\sqrt{\frac{1}{N}\sum_{i=1}^{N}n_{i}{\left(\vec{r_{i}}-\vec{r_{cm}}\right)}^{2}},$$ (4)

to characterize the typical distance occupied by an individual’s trajectory. Here *N* is the total number of the distinct locations, $\vec{r_{i}}$ is the geographic coordinates of location *i = 1*, *2*, *…*, *N*, *n*_*i*_ is the visit frequency or the waiting time in location *i* and $\vec{r_{cm}}=1/N\sum_{i=1}^{N}n_{i}\vec{r_{i}}$ represents the center of mass of the trajectory. When computing the radius of gyration, usually we can set *n*_*i*_ as visit frequency for low sampling rate and waiting time for high sampling rate and the computation result of *r*_*g*_ varies when the meaning of *n*_*i*_ differs. Since the time scales ranged from 7.5 min to 32 hours in our experiment, we used visit frequency as the weight *n*_*i*_ of location *i* for consistency. In addition, when we studied how radius of gyration changed over time, we used *r*_*g*_*(T)* to represent the gyration radius to time *T* and *N* and $\vec{r_{cm}}$ changed to *N(T)* and $\vec{r_{cm}(T)}$ accordingly.

### Computation of waiting time

Due to the sparsity of CDR, when computing the distribution for waiting time on a CDR data set, we have to discretize the time series with a unit *T* and find a cell tower of a CDR record for each interval[21]. The limitations of this method lie in the large *T* (typically 1 or 2 hours) and massive intervals without location information. MFRs have finer-grained temporal resolution and can overcome these two defects. When computing waiting time, we first sorted an individual’s MFRs in time order and then combined consecutive records if they had the same cell tower ID. Waiting time at each cell tower could be computed by subtracting the first timestamp from the last one at this tower.

### Trajectory visualization

We visualized the trajectories on the map in order to help to analyze human mobility. The map shown in Fig 1 was generated with Mapbox GL JS (https://www.mapbox.com/mapbox-gl-js/api/) and map data by OpenStreetMap contributors (License: https://opendatacommons.org/licenses/odbl/). Trajectories are visualized with D3JS (https://d3js.org/). An interactive website for trajectory visualization can be found at **Data Availability**.

## Supporting information

S1 Fig Displacement of human mobility (D1). **A-D,** The distribution of displacement *P(Δr)* and its best fits under time scales *δ* ≈ 15min(n = 8,258,692), 1hour(n = 5,071,176), 4hour(n = 2,226,450), 16hour(n = 702,218), respectively. The solid lines (green and blue) indicate a truncated power law and a log-normal distribution with best fitting parameters. The insets show the best power law fitting for the tails (from 70%ile to 96%ile).
(PDF)

S2 Fig Displacement of human mobility (D2). **A**, The distribution of displacement *P(Δr)* under time scales *δ* ≈ 7.5min(n = 7,567,233), 30min(n = 7,006,878), 2hour(n = 3,498,286), 8hour(n = 1,347,365), 32hour(n = 508,482). **B-J**, The distribution of displacement *P(Δr)* and its best fits under time scales *δ* ≈ 7.5min, 15min(n = 6,991,657), 30min, 1hour(n = 6,457,261), 2hour, 4hour(n = 4,259,369), 8hour, 16hour(n = 1,880,823), 32hour, respectively. The solid lines (green and blue) indicate a truncated power law and a log-normal distribution with best fitting parameters, respectively. The insets show the best power law fitting for the tails (from 70%ile to 90%ile). **K.** The variation trend of fitting parameter *β* with time scale *δ* with standard deviation as error bar.
(PDF)

S3 Fig Gyration radius of human mobility (D1). **A**, The distribution of gyration radius *r*_*g*_ under time scales *δ* ≥ 7.5min, 30min, 2hour, 8hour, 32hour(n = 142,619). We further divide all users into 3 groups (n = 5,001, 3,198 and 2,217) according to their final gyration radius *r*_*g*_*(T)* during the whole observation period *T*. **B-D,** show the convergence speeds of *r*_*g*_ of different user group *r*_*g*_*(T)* = *R*_*g*_ ± 0.05*R*_*g*_ and *R*_*g*_ = 5km, 10km, 15km, respectively.
(PDF)

S4 Fig Time related statistics of human mobility (D2). **A**, The distribution of waiting time *Δt* under time scales *δ*_*min*_(n = 66,821,244) and *δ* ≥ 7.5min(n = 38,617,952), 30min(n = 25,859,924), 2hour(n = 16,329,587), 8hour(n = 9,132,801) and 32hour(n = 3,882,266). **B**, The distribution of moving speed under time scales *δ* ≈ 7.5min(n = 5,249,717), 30min(n = 7,281,423), 2hour(n = 5,400,576), 8hour(n = 3,112,735), 32hour(n = 1,666,615). **C,** The distribution of radius of gyration for 3 user groups with different max speeds 0 < *v*_*max*_ ≤ 6km/h (n = 13,807), 15 < *v*_*max*_ ≤ 25km/h (n = 22,516) and 30 < *v*_*max*_ ≤ 60km/h (n = 38,473) during the whole observation period *T*. **D,** The distribution of moving distance for each group during morning rush hour (7am to 10am).
(PDF)

S5 Fig *KS* test for Fig 3 and S1 Fig. **A-I**, The *KS* test result of the best power law fitting for the tail distribution of displacement under time scales *δ* ≈ 7.5min, 15min, 30min, 1hour, 2hour, 4hour, 8hour, 16hour and 32hour. The fits under all time scales passed the *KS* test except for 16hour due to the roughness at *Δr* ≈ 25km.
(PDF)

S6 Fig *KS*_*W*_ test for Fig 3 and S1 Fig. **A-I**, The *KS*_*W*_ test result of the best power law fitting for the tail distribution of displacement under time scales *δ* ≈ 7.5min, 15min, 30min, 1hour, 2hour, 4hour, 8hour, 16hour and 32hour. The fits under all time scales passed the *KS*_*W*_ test.
(PDF)

S1 Table Columns of Mobile Flow Records (MFRs). Detailed description for some key columns of MFRs about human behavior.
(PDF)
