# Supplementary figures and images for: Temporal understanding of human mobility: A multi-time scale analysis
Source: PLoS One. 2018 Nov 27;13(11):e0207697. doi: 10.1371/journal.pone.0207697 (PMC6258540; doi:10.1371/journal.pone.0207697)

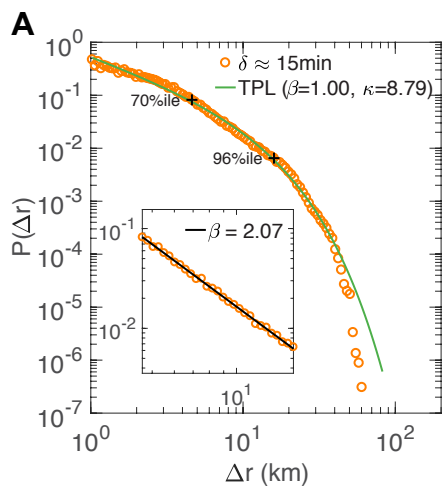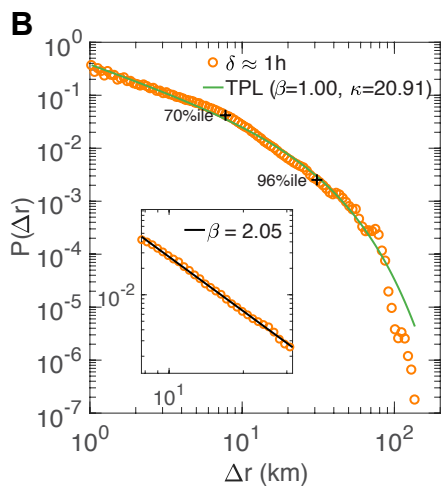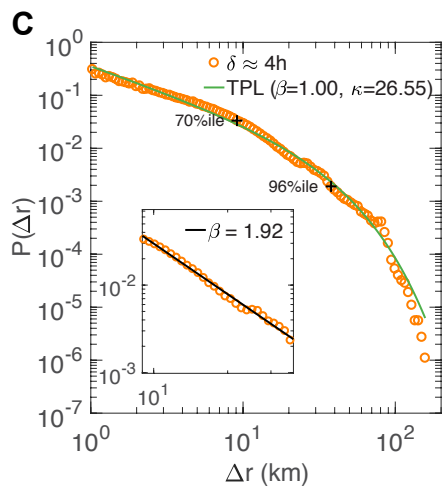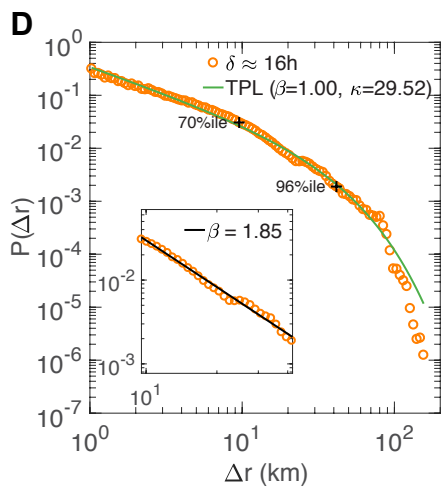

Supplement: S1 Fig — A-D, The distribution of displacement P(Δr) and its best fits under time scales δ ≈ 15min(n = 8,258,692), 1hour(n = 5,071,176), 4hour(n = 2,226,450), 16hour(n = 702,218), respectively. The solid lines (green and blue) indicate a truncated power law and a log-normal distribution with best fitting parameters. The insets show the best power law fitting for the tails (from 70%ile to 96%ile). (PDF) [file pone.0207697.s001.pdf]

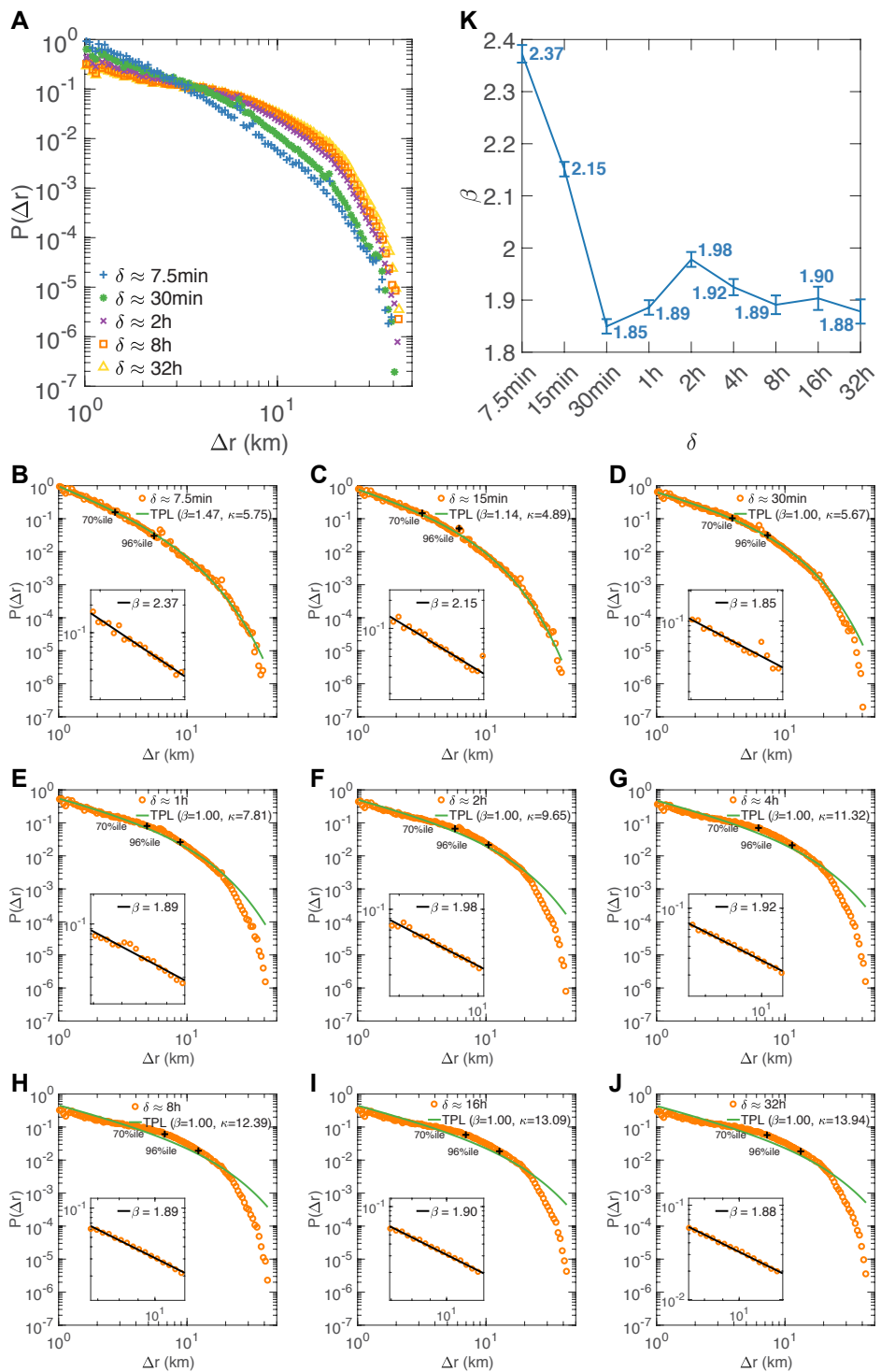

Supplement: S2 Fig — A, The distribution of displacement P(Δr) under time scales δ ≈ 7.5min(n = 7,567,233), 30min(n = 7,006,878), 2hour(n = 3,498,286), 8hour(n = 1,347,365), 32hour(n = 508,482). B-J, The distribution of displacement P(Δr) and its best fits under time scales δ ≈ 7.5min, 15min(n = 6,991,657), 30min, 1hour(n = 6,457,261), 2hour, 4hour(n = 4,259,369), 8hour, 16hour(n = 1,880,823), 32hour, respectively. The solid lines (green and blue) indicate a truncated power law and a log-normal distribution with best fitting parameters, respectively. The insets show the best power law fitting for the tails (from 70%ile to 90%ile). K. The variation trend of fitting parameter β with time scale δ with standard deviation as error bar. (PDF) [file pone.0207697.s002.pdf]

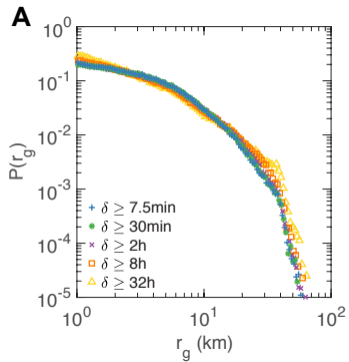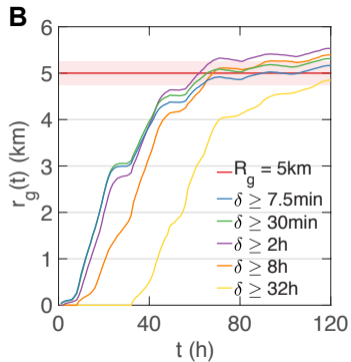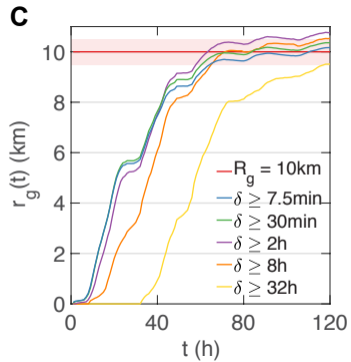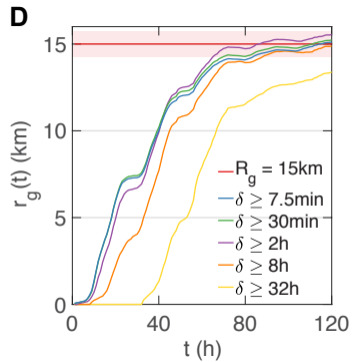

Supplement: S3 Fig — A, The distribution of gyration radius rg under time scales δ ≥ 7.5min, 30min, 2hour, 8hour, 32hour(n = 142,619). We further divide all users into 3 groups (n = 5,001, 3,198 and 2,217) according to their final gyration radius rg(T) during the whole observation period T. B-D, show the convergence speeds of rg of different user group rg(T) = Rg ± 0.05Rg and Rg = 5km, 10km, 15km, respectively. (PDF) [file pone.0207697.s003.pdf]

**A**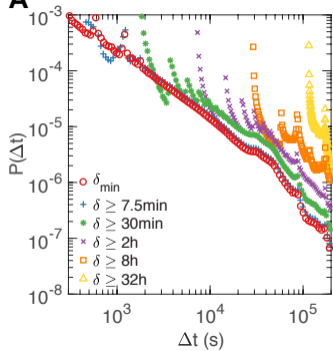**B**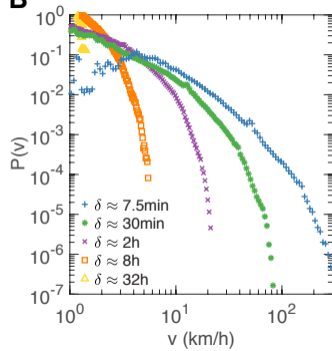**C**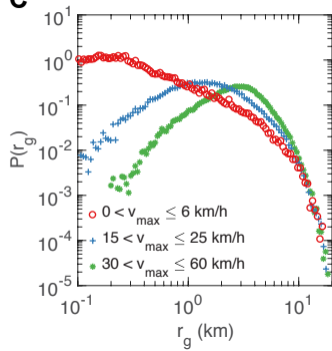**D**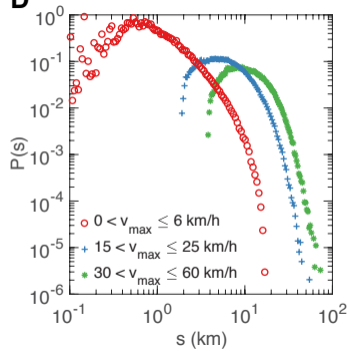

Supplement: S4 Fig — A, The distribution of waiting time Δt under time scales δmin(n = 66,821,244) and δ ≥ 7.5min(n = 38,617,952), 30min(n = 25,859,924), 2hour(n = 16,329,587), 8hour(n = 9,132,801) and 32hour(n = 3,882,266). B, The distribution of moving speed under time scales δ ≈ 7.5min(n = 5,249,717), 30min(n = 7,281,423), 2hour(n = 5,400,576), 8hour(n = 3,112,735), 32hour(n = 1,666,615). C, The distribution of radius of gyration for 3 user groups with different max speeds 0 < vmax ≤ 6km/h (n = 13,807), 15 < vmax ≤ 25km/h (n = 22,516) and 30 < vmax ≤ 60km/h (n = 38,473) during the whole observation period T. D, The distribution of moving distance for each group during morning rush hour (7am to 10am). (PDF) [file pone.0207697.s004.pdf]

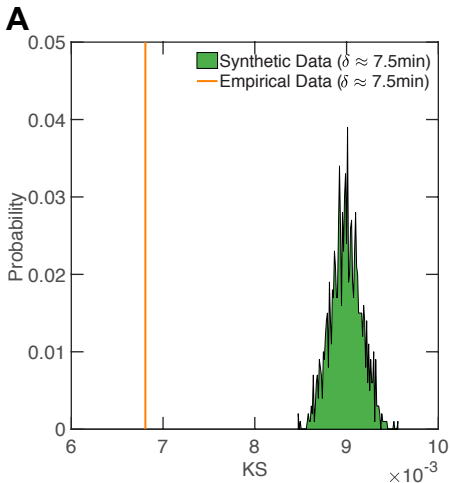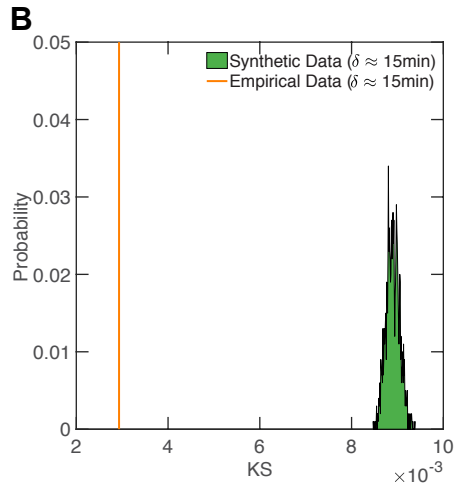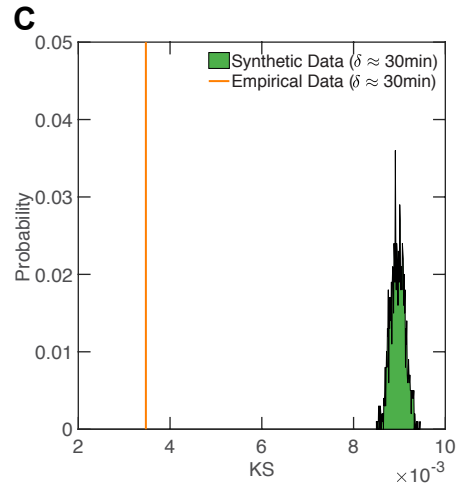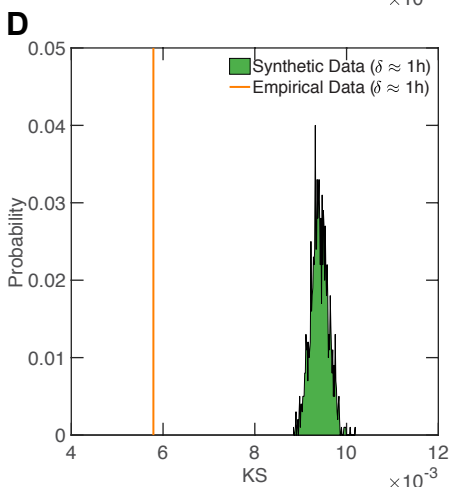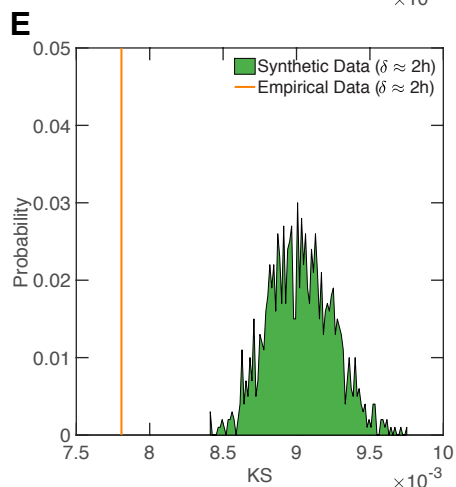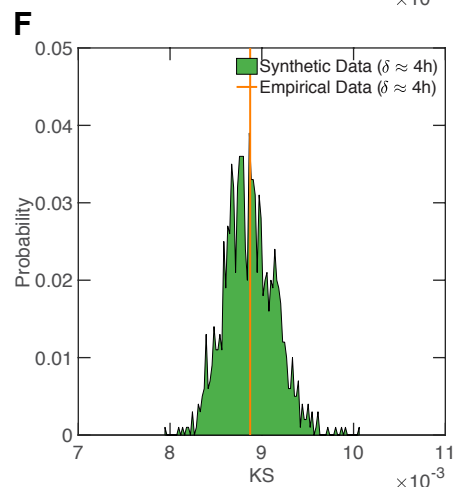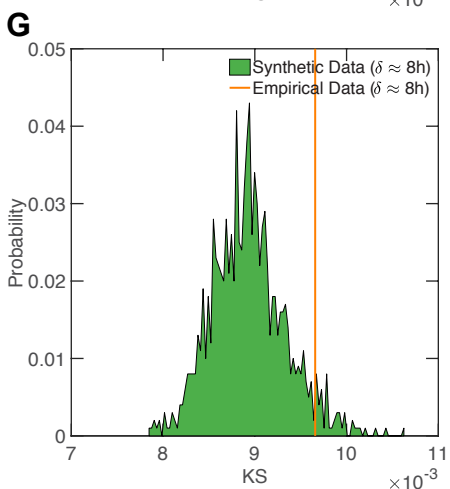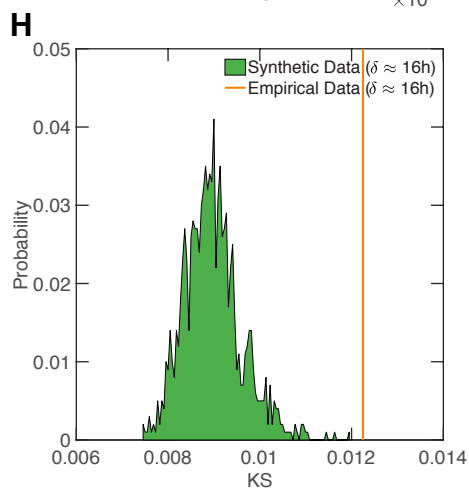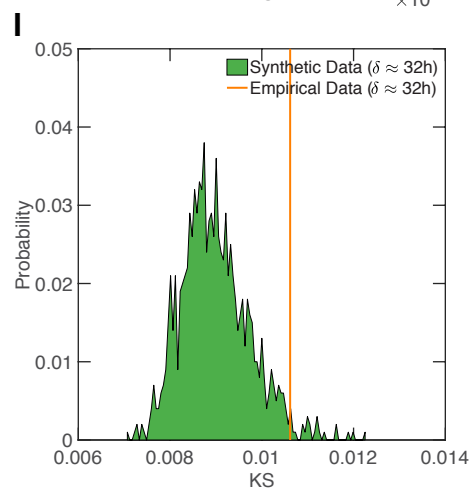

Supplement: S5 Fig — A-I, The KS test result of the best power law fitting for the tail distribution of displacement under time scales δ ≈ 7.5min, 15min, 30min, 1hour, 2hour, 4hour, 8hour, 16hour and 32hour. The fits under all time scales passed the KS test except for 16hour due to the roughness at Δr ≈ 25km. (PDF) [file pone.0207697.s005.pdf]

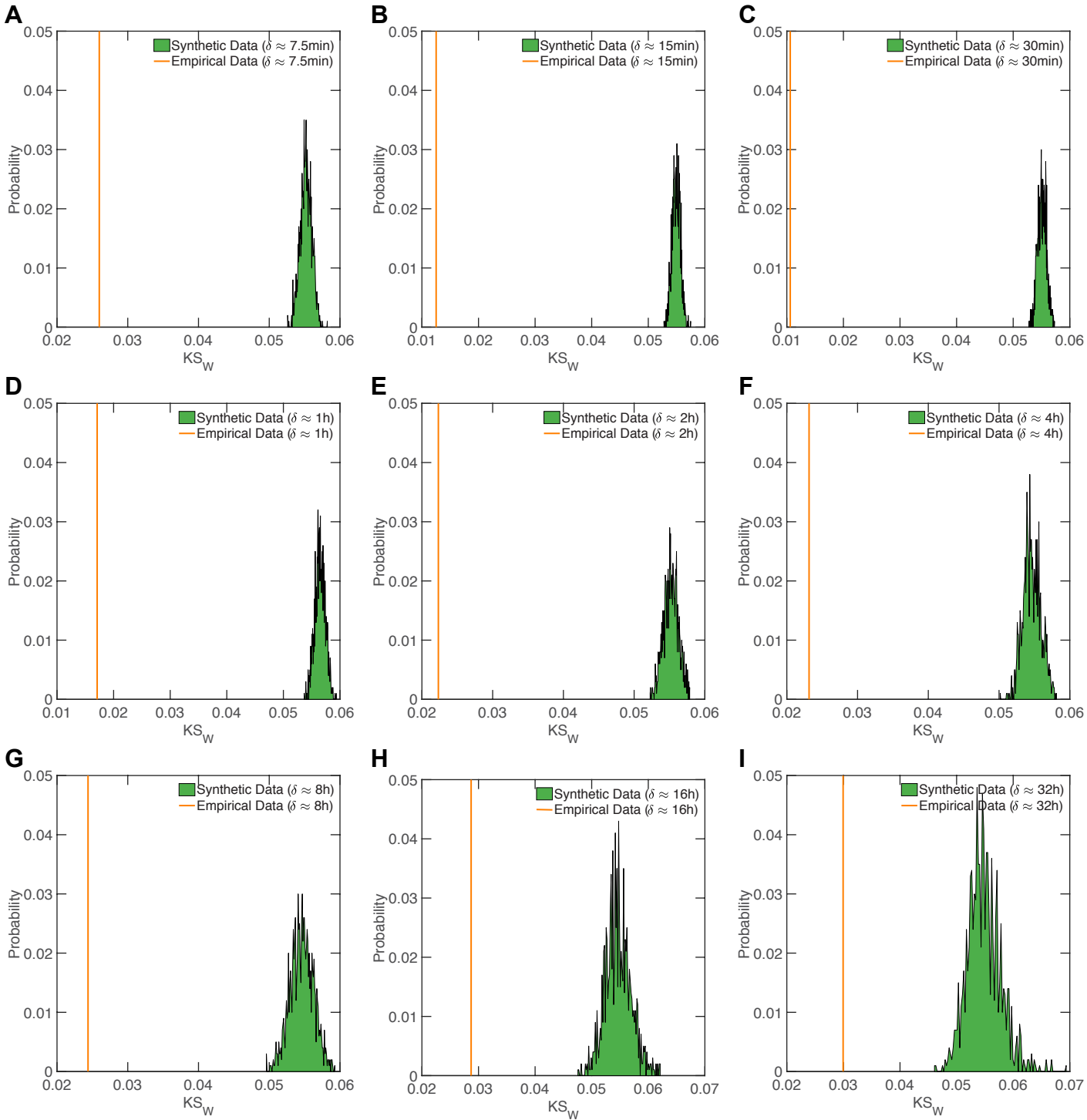

Supplement: S6 Fig — A-I, The KSW test result of the best power law fitting for the tail distribution of displacement under time scales δ ≈ 7.5min, 15min, 30min, 1hour, 2hour, 4hour, 8hour, 16hour and 32hour. The fits under all time scales passed the KSW test. (PDF) [file pone.0207697.s006.pdf]
