# Supplementary material for: Temporal understanding of human mobility: A multi-time scale analysis
Source: PLoS One. 2018 Nov 27;13(11):e0207697. doi: 10.1371/journal.pone.0207697 (PMC6258540; doi:10.1371/journal.pone.0207697)
Supplement: S1 Table — Detailed description for some key columns of MFRs about human behavior. (PDF) [file pone.0207697.s007.pdf]

Temporal understanding of human mobility: a multi-time scale analysis
Supporting Tables

Tongtong Liu[1,¶], Zheng Yang[1,*,¶], Yi Zhao[1], Chenshu Wu[2], Zimu Zhou[3], Yunhao Liu[1]

[1]Tsinghua University, Beijing, 100084, China.
[2]University of Maryland, College Park, USA.
[3]Swiss Federal Institute of Technology in Zurich, Zurich, Switzerland.

*Corresponding author
Email: yangzheng@tsinghua.edu.cn

¶These authors contributed equally to this work.

**Column Name**	**Description**
createtime	Timestamp of creation time
flow_id	Unique ID of a flow
orig_id	Unique ID of a user
orig_device	Device Type ID
orig_bs	Connected Cell Tower ID
orig_ip	IP Address of the Client
orig_port	Port of the Client
application	Application ID
flow_start	Start time of a flow
flow_end	End Time of a flow
os	Device OS ID
http_host	HTTP host
http_uri	HTTP uri
…	other information

S1 Table. Columns of Mobile Flow Records (MFRs). Detailed description for some key columns of MFRs about human behavior.
